# Unveiling a Unique Presentation of Superior Vena Cava Syndrome Succeeding a Traumatic Motor Vehicle Crash and Implantable Cardioverter-Defibrillator Lead Placement

**DOI:** 10.7759/cureus.61303

**Published:** 2024-05-29

**Authors:** Jenna Sapone, Mathai Chalunkal

**Affiliations:** 1 Internal Medicine, St. Luke's University Health Network, Easton, USA

**Keywords:** cardiac tamponade, pericardial effusion, superior vena cava (svc) obstruction, svc obstruction, thrombosis, blunt cardiac trauma, icd lead, superior vena cava (svc) syndrome

## Abstract

Superior vena cava (SVC) syndrome, once a rarity, has seen an uptick in cases with diverse origins. While this disease process is clinically diagnosable, imaging modalities and tissue biopsies further refine interventions. The clinical presentation includes but is not limited to edema of the arms, neck, and head, facial plethora, cyanosis, and or distention of subcutaneous vessels. SVC syndrome can be attributed to extrinsic compression or thrombosis in many cases. If symptoms are not life-threatening, the overall morbidity is based on the underlying root cause. Few cases have been reported with associated death due to epistaxis. However, the obstruction itself can be initially asymptomatic and then slowly progress over months to years. This case report highlights a distinct instance of SVC syndrome with notable risk factors: implantable cardioverter defibrillator placement and prior cardiac trauma status post-intervention.

## Introduction

Superior vena cava (SVC) syndrome was first discovered in 1757 by William Hunter, initially linked with large syphilitic aortic aneurysms. During that time, there were two leading causes, which included tuberculosis and syphilis. Nearly 300 modern cases have been reported, however primarily stemming from mediastinal malignancies [1]. More recent research identifies increased thrombotic events due to intravascular catheterization and device implantations. In the United States, around 15,000 cases of SVC syndrome are reported annually. Prognosis is dependent on the etiology of obstruction, and worse outcomes are associated with malignancy. Unfortunately, malignancy now accounts for the majority of SVC obstructions. Iatrogenic injuries are common and can be diagnosed pre-operatively, and the proper surgical approach was chosen accordingly. In contrast, penetrating SVC injuries are rare but highly lethal. However, prospective studies should be considered on those survivors to risk stratify and monitor for progression to SVC syndrome. To advance as interventionalists, management strives for new techniques that improve outcomes. However, some therapies include straightforward oral anticoagulation. This case may open many avenues to delineate risk factors, particularly for patients who are at higher than average risk, for the development of SVC obstruction. We present a case entwining cardiac trauma, implantable cardioverter-defibrillator (ICD) insertion, and SVC syndrome.

## Case presentation

**Figure 1:**
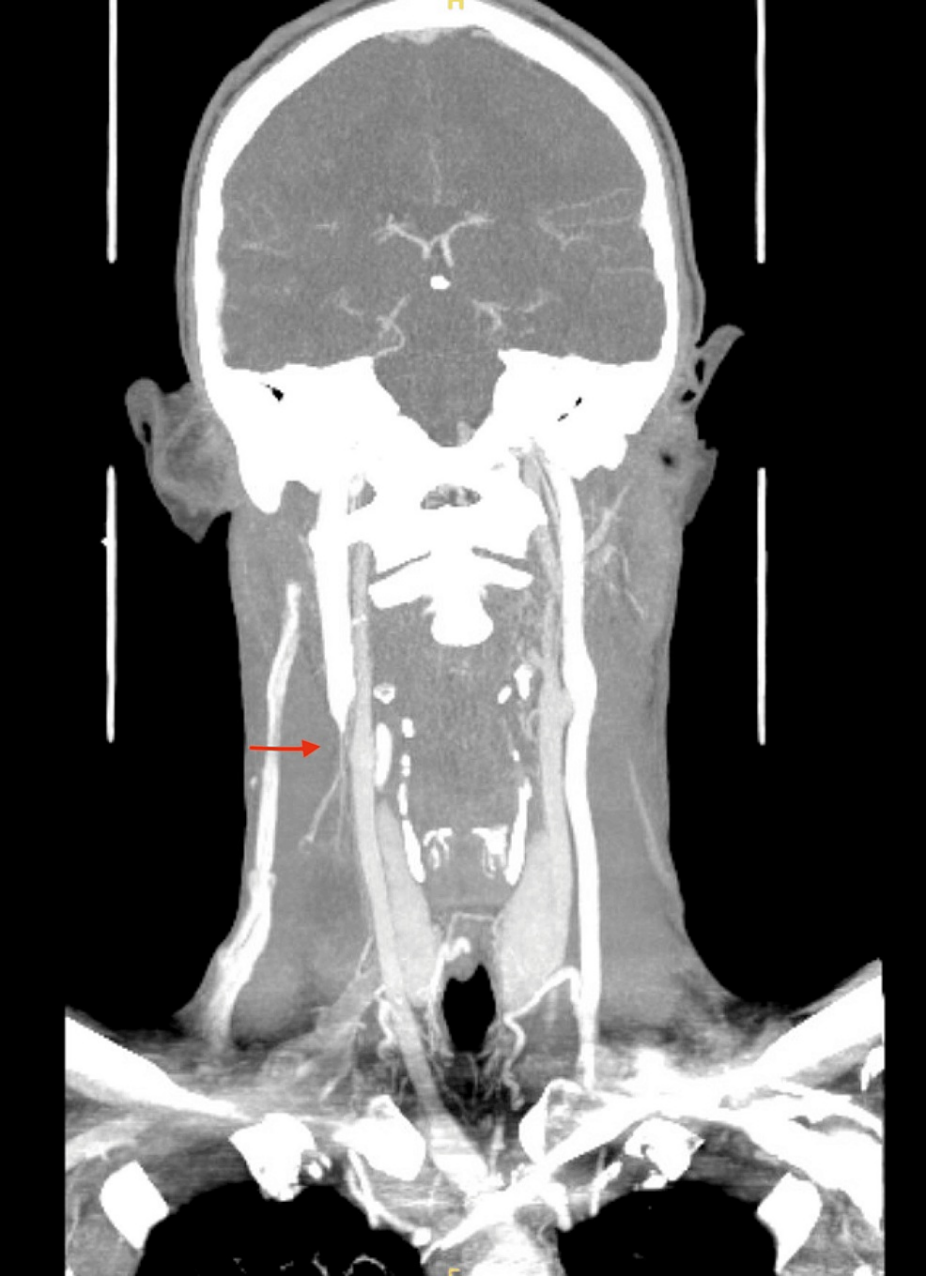
CT venogram of the head and neck The red arrow depicts an occlusive thrombus of the right internal jugular vein, which extends from the level of C3 inferiorly to the right brachiocephalic vein, superior vena cava above the level of the azygos vein, and occluded right subclavian vein.

**Figure 2:**
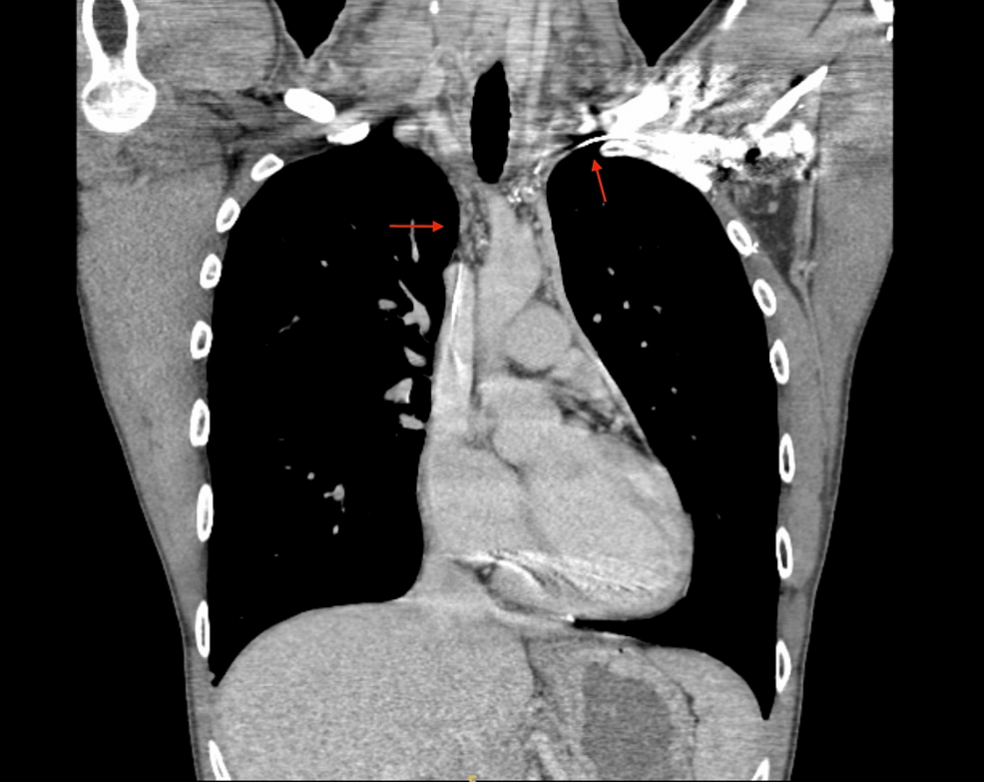
CT venogram of the chest The red arrow on the right depicts an occlusive thrombus of the right internal jugular vein with chronic total occlusion of the superior vena cava above the level of the azygos vein, along with occlusion of the right brachiocephalic vein. This also extends into chronic occlusions of the left brachiocephalic vein, which contains an automatic implantable cardioverter-defibrillator wire, indicated by the left red arrow.

A 24-year-old male, with a past medical history of long QT syndrome, ICD placement, and tobacco and marijuana abuse, presented to the emergency department due to altered mental status. Several hours before arrival, the patient was involved in a motor vehicle accident and did not seek medical attention. The patient decompensated acutely and was found to have acute cardiac tamponade necessitating mediastinal exploration. Exploration revealed a large pericardial effusion with pericardial tamponade, along with a perforation of the right atrium at the junction of the superior vena cava. Five years later, the patient presented to his primary care physician (PCP) with complaints of intermittent swelling of his neck and face, along with occasional dizziness and blurred vision. Computed tomography (CT) venogram of the head, neck, and chest demonstrated an occlusive thrombus of the right internal jugular vein into the right brachiocephalic, right subclavian, and SVC, along with occlusion of the left brachiocephalic vein containing the left subclavian approach of the cardiac defibrillator wire, as well as an old occluded left subclavian vein. Fortunately, collateral flow was noted in the azygous veins, and the superior vena cava just below the thrombus (Figure 1 and Figure 2). The patient underwent multiple vascular surgery evaluations. With his significant cardiac history and reconstruction, he was at high risk for any surgical intervention; therefore, oral anticoagulation was preferred.

## Discussion

SVC obstruction syndrome is defined as the stenosis or occlusion of the SVC, leading to obstruction of the venous outflow of the head and upper extremities. The obstruction of the SVC can be caused by a multitude of conditions; however, they can be broken down into the most common differentials, including malignancy with extrinsic compression, infection, indwelling catheters or central venous access devices, thrombosis, and/or trauma. SVC obstruction has a typical clinical presentation, most notably for swelling of the upper face, distention of veins across the chest, upper arms, neck, shortness of breath, cyanosis, and plethora. Other typical clinical manifestations also include dizziness, blurry vision, cough, hoarseness, dyspnea, and even syncopal episodes.

Clinical evaluation should always begin with a full history and physical examination, with much attention on the history of malignancy or invasive procedures. The urgency of intervention has not been well established based on particular criteria. However, a recent study [2] created a classification system that may be utilized to perform a stratification of various symptoms and grades, as seen in Table 1. It has been proposed that the use of this schema in future studies should provide a common language to describe the patients and thereby help define the role of interventions [2]. This schema is patterned after the Common Terminology Criteria for Adverse Events (CTCAE) of the National Institutes of Health. This categorization does not address SVC syndrome, although it does include a category of edema of the head and neck. The grading in this CTCAE category is similar to the proposed schema, except that the CTCAE is more narrowly focused, whereas the proposed schema includes all symptoms caused by SVC obstruction [2].

**Table 1:** Grading system for the superior vena cava syndrome Above is a proposed classification system that was created to assess the severity of symptoms to determine the urgency of intervention, which included grade, category, estimated incidence, and definition [2].

Grade	Category	Estimated Incidence (%)	Definition
0	Asymptomatic	10	Radiographic superior vena cava obstruction in the absence of symptoms
1	Mild	25	Edema in the head or neck (vascular distension), cyanosis, plethora
2	Moderate	50	Edema in the head or neck with functional impairment (mild dysphagia, cough, mild or moderate impairment of the head, jaw, or eyelid movements, visual disturbances caused by ocular edema)
3	Severe	10	Mild or moderate cerebral edema (headache, dizziness) or mild/moderate laryngeal edema or diminished cardiac reserve (syncope after bending)
4	Life-Threatening	5	Significant cerebral edema (confusion, obtundation) or significant laryngeal edema (stridor) or significant hemodynamic compromise (syncope without precipitating factors, hypotension, renal insufficiency)
5	Fatal	<1	Death

In addition to clinical evaluation, imaging is the next step in management, which could also be diagnostic. Methods include chest X-rays, which could demonstrate mediastinal widening, the most common sign in approximately 60% of patients. On the contrary, chest radiographs can also be normal. CT chest with contrast can demonstrate the level of obstruction and the development of collateral vessels and/or can identify the cause of obstruction [3]. Bilateral arm venography could potentially exhibit the presence of obstruction, but it has limitations and may overestimate the level of obstruction due to shunting into collateral vessels. An alternative to venography would be direct venography. Histological diagnoses can be ascertained through sputum cytology, bronchoscopy, fine needle aspiration, and/or CT-guided biopsies.

Further discussion includes various etiologies of SVC syndrome, including typical presentations, management, and treatment options.

Malignancy and infection 

Commonly SVC syndrome can initially present as an extrinsic compression secondary to an undiagnosed tumor in up to 70% of cases. Non-small cell lung cancers (NSCLC) account for around 50% of the cases, small cell lung cancer (SCLC) is around 25%, and around 10% are due to lymphomas [4]. Following those imaging modalities and biopsies, treatment approaches are multidisciplinary in this setting. Management is guided by the severity of symptoms with a histologic diagnosis of biopsied tissue. A general algorithm used to guide treatment begins with whether the SVC syndrome is malignant. If it is not, as directly by the cause, one would utilize anticoagulation or antibiotics as appropriate. If the findings are malignant, immediate stenting is used for symptom palliation [5]. After a histologic diagnosis is found, either therapy with radiation and/or chemotherapy is indicated. Chemo-sensitive diseases, including SCLC, non-Hodgkin lymphoma, and germ cell tumors, are considered the mainstay of treatment [3]. Radiation therapy with or without chemotherapy is the mainstay of treatment for most patients. As previously stated, intra-vascular stents are proven to be safe and effective and allow the most rapid resolution of symptoms. Because the overall prognosis of the majority of patients with malignancy is poor, palliation is often the focus of treatment. Infections have been incidentally discovered as another primary cause of SVC syndrome, though it does happen rarely. SVC obstruction secondary to infection, including cases of tertiary syphilis, melioidosis, histoplasmosis, actinomycosis, and tuberculosis, has been, at large, the most reported cause. It has been reported that those with implantable cardiac devices are at higher risk, however overall, this can be difficult to diagnose. A case report was written due to an unusual presentation of pacemaker lead-associated endocarditis initially presented due to facial flushing with exertion found to be due to SVC obstruction. Pacemaker-lead infection has been implicated in the pathogenesis of SVC obstruction by inducing inflammation and subsequent fibrosis and through mechanical obstruction by thrombus formation [6]. Treatment is typically guided by the offending infection with antibiotics and/or surgical intervention if source control is required.

Indwelling catheters, central venous access devices, and thrombosis 

Approximately 300,000 ICDs and 500,000 pacemakers are implanted in the United States per year. Cardiac-implanted devices, such as pacemakers and ICDs with indwelling leads, are a common consequence of venous obstruction. Venous obstruction of the subclavian, brachiocephalic, or SVC are common vessel complications due to the implanted leads. This complication is often asymptomatic but is discovered when attempting to insert new leads. Symptoms typically range from mild swelling to morbid venous congestion associated with SVC syndrome. Pathophysiology comprises lead-associated venous narrowing and occlusion due to thrombosis and fibrosis. Early after the lead is inserted into the vein, there is an early prothrombic reaction resulting from endothelial damage and perturbation of blood flow. The endothelial injury and presence of a foreign body lead to an inflammatory cascade. Circulating procoagulant factors also increase soon after lead placement [7]. Significant occlusion is defined as >50% vessel narrowing seen in around 30% of patients and complete occlusion in about 10%. SVC syndrome secondary to lead-related obstruction has been reported in about 30% of patients [7]. Clinical presentation can vary but may include ipsilateral arm swelling that correlates with the occlusion of brachiocephalic, subclavian, or axillary veins. Some patients may even present with pain from collateral flow, which can be on the chest wall, shoulders, or subclavicular regions. It has been reported that, in acute cases within two days of symptom onset, treatment incorporates thrombolytic therapy, mechanical thrombectomy, or catheter-directed thrombolysis, followed by anticoagulation. For thrombosis that is greater than 10 days of symptom onset, it has been shown that the above-listed therapies are less effective due to the organization of the thrombus itself [1]. Anticoagulation remains the mainstay of treatment, and thrombus debulking, lead extraction, venoplasty, and stenting are all important therapeutic interventions [7].

Trauma 

There are rare incidences of SVC syndrome due to trauma that have been reported. A total of 13 cases occurred after aortic or cardiac surgery and four after blunt chest trauma. A few of these patients demonstrated frank SVC syndrome; the others experienced symptoms compatible with SVC syndrome but not exclusive to it (e.g., dyspnea) [8]. Interestingly, none of these presentations were acute, and the minimum time from “injury” to presentation was three months [8]. Research also demonstrates that presentations of SVC syndrome may be incidental in the setting of trauma, which stems from a chronic etiology. Retrospective studies should be performed in these cases due to the long-term insidious presentations that occur months to years later.

## Conclusions

This case underscores the evolving landscape of SVC syndrome etiologies and interventions. Recognition of at-risk patients, advances in antithrombotic measures, and vigilance regarding past trauma are imperative for comprehensive management. By dissecting common differentials, presentations, and treatment modalities, this report contributes to the collective understanding and management of SVC syndrome.
